# Innovative Sol-gel functionalized polyurethane foam for sustainable water purification and analytical advances

**DOI:** 10.3389/fchem.2024.1324426

**Published:** 2024-02-08

**Authors:** H. Alwael, A. N. Alsulami, T. N. Abduljabbar, M. Oubaha, M. S. El-Shahawi

**Affiliations:** ^1^ Department of Chemistry, Faculty of Science, King Abdulaziz University, Jeddah, Saudi Arabia; ^2^ Centre for Research in Engineering Surface Technologies (CREST), FOCAS Institute, Technological University Dublin, Dublin, Ireland

**Keywords:** environmental cleaning, kinetics and thermodynamics, retention mechanism, reusability, Sol-gel/polyurethane foams sorbent

## Abstract

Nanomaterial combined polymeric membranes such as polyurethane foams (PUFs) have garnered enormous attention in the field of water purification due to their ease of management and surface modification, cost-effectiveness, and mechanical, chemical, and thermal properties. Thus, this study reports the use of novel Sol-gel impregnated polyurethane foams (Sol-gel/PUFs) as new dispersive solid phase microextractors (d- µ SPME) for the efficient separation and subsequent spectrophotometric detection of Eosin Y (EY) textile dye in an aqueous solution with a pH of 3–3.8. The Sol gel, PUFs, and Sol gel–impregnated PUFs were characterized using scanning electron microscopy (SEM), goniometry measurements, dynamic light scattering (DLS), energy dispersive spectroscopy (EDS), UV-Visible, and FTIR spectra. Batch experiment results displayed a remarkable removal percentage (96% ± 5.4%) of the EY from the aqueous solution, with the total sorption time not exceeding 60 min. These data indicate rate-limited sorption via diffusion and/or surface complex ion associate formations after the rapid initial sorption steps. A pseudo-second order kinetic model thoroughly explained the sorption kinetics, providing a sorption capacity (q_e_) of 37.64 mg g^−1^, a half-life time (t_1/2_) of 0.8 ± 0.01 min, and intrinsic penetration control dye retention. The thermodynamic results revealed a negative value for ΔG^⁰^ (−78.07 kJ mol^−1^ at 293 K), clearly signifying that the dye uptake was spontaneous, as well as a negative value for ΔH^⁰^ (−69.58 kJ mol^−1^) and a positive value for ΔS^⁰^ (147.65 J mol^−1^ K^−1^), making clear the exothermic nature of EY adsorption onto the sorbent, with a growth in randomness at the molecular level. A ternary retention mechanism is proposed, involving the “weak base anion exchanger” of {(–CH_2_–OH^+^ –CH_2_–) (Dye anion)^−^}_Sol-gel_/_PUF_ and/or {(–NH_2_^+^ –COO-) (Dye anion)^−^}_Sol-gel_/_PUF_ via solvent extraction and “surface adsorption” of the dye anion on/in the Sol-gel/PUFs membranes in addition to H-bonding, including surface complexation and electrostatic π-π interaction, between the dye and the silicon/zirconium oxide (Si-O-Zr) and siloxane (Si-O-Si) groups on the sorbent. Complete extraction and recovery (93.65 ± 0.2, −102.28 ± 2.01) of EY dye with NaOH (0.5 M) as a proper eluting agent was achieved using a sorbent-packed mini column. In addition, the established extractor displayed excellent reusability and does not require organic solvents for EY enrichment in water samples, making it a talented nominee as a novel sorbent for EY sorption from wastewater. This study is of great consequence for expanding the applicatio1n of Sol-gel/PUFs in developing innovative spectrophotometric sensing strategies for dye determination. In view of this, it would also be remarkable to perform future studies to explore the analytical implications of this extractor regarding safety and environmental and public health issues associated to the pollutant.

## 1 Introduction

Nowadays, with the advancement of industrialization, the global demand for fresh water is expected to increase by 55% between 2000–2050 (LiShi et al., 2023). Water crises arising from the release of industrial effluents into fresh water without treatment have attracted global attention and condemnation (Egbosiuba et al., 2022). Thus, widespread pollution of water resources dares researchers to combat this environmental crisis by devising and applying befitting technologies. Industrial development has led to many disturbing effects on nature, and globally, textile industries are playing a vital role in generating effluent (Egbosiuba et al., 2022; LiShi et al., 2023; Ma et al., 2023). Textile dyes in water can enter the human body through the food chain, leading to irreversible damage to human health (Egbosiuba et al., 2022; LiShi et al., 2023; Ma et al., 2023). The rapid and sensitive identification or capture of organic/inorganic pollutants is of great consequence for protecting ecosystems and human health (Tang et al., 2023). In surface and groundwater samples, the concentration of various pollutants increases due to various worldwide anthropogenic accomplishments. The majority of these contaminants have become a severe concern and can generate a massive, unsettling health and public worry for aquatic organisms (Tran et al., 2019). The world is also currently confronted with huge hurdles in supplying the growing request for unpolluted water since accessible supplies of fresh water are rapidly exhausting (Saigl et al., 2023).

Today, water pollution by dye industries represents one of the chief challenges that affects the whole world, mainly a result of the incompetence of textile operations to properly dispose of their waste in water (Annan et al., 2021). Textile industries are major contributors to the global economy as well as environmental pollution in China, South African estuaries, and many countries (Abdul Mubarak et al., 2020). Wastewater containing dyes is a significant polluter of the aquatic environment, and also affects human health, as textile industries generate large amounts of highly coloured wastewater containing dyes (World Health Organization, 1996; Abdul Mubarak et al., 2020). Textile dyes at micro and ultra-micro levels are among the most hazardous and highly harmful contaminants in the aquatic environment, where approximately 5,000 tons of dyes are unconstrained every year in the aquatic environment (World Health Organization, 1996). Thus, it is very crucial to develop a highly effective and sensitive solid extractor for textile dyes to solve the major problem in environment health.

Most textile dyes have a high-water solubility and a slow rate of natural degradation, which allows them to remain in the environment for a long time (Pirkarami and Olya, 2017; Azadfara et al., 2021; Babazadeh et al., 2021). Therefore, the development of fast and reliable solid phase extractors by environmental experts has become a major global challenge for environmental protection, and removing textile dyes from harmful effluents including wastewater has become a major global challenge for environmental protection (Robinson et al., 2001; Baharak and Ali, 2013). Eosin yellow (EY) dye (Electronic Supplementary Information, ESI 1) may cause severe skin and eye irritation (Pavlidis et al., 2003; Bassey et al., 2012). Direct contact of Eosin yellow dye with the eye can also be a source of eternal damage to the cornea by destroying retinal ganglion cells located near the retina inner surface (Sun et al., 1987; Borzelleca et al., 1987). Thus, great attention has been paid toward minimization and/or complete removal of textile dyes from aquatic environmental wastewater using various extraction techniques (Baharak and Ali, 2013; Ayati et al., 2019).

In today’s era, according to the importance of water treatment, great attempts have been explored in the adsorption removal process via synthesis and functionalization of various adsorbents such as ionic liquid-modified composites (Ayati et al., 2019), modified xanthan gum/silica hybrid nanocomposites (Ghorai et al., 2013), chitosan-based adsorbents (Bahrudin and Nawi, 2019; Bahrudin et al., 2020; Karimi-Maleh et al., 2021), spent tea leaves (Hameed, 2009), and polyurethane foams (PUFs), with the aim of improving their adsorption behaviour toward textile dyes and other organic pollutants (El-Shahawi, 1994; Neta et al., 2011; Han et al., 2017; El-Shahawi and Alwael, 2019). Sol-gel technology has also aided in the development of a significant variety of innovative solid sorbents with enormous surface areas, good thermal and solvent stability, and high selectivity (Kabir et al., 2013; Amiri, 2016; Kabir et al., 2016; Tabesh et al., 2018). Solid-phase microextraction based on Sol-gel techniques offers a rapid, easy, and suitable pathway to prepare chemically bonded, stable, and dispersive solid phase microextractor (d-µ-SPME) coatings (Kabir et al., 2013; Tabesh et al., 2018). The most common techniques involving the use of Sol-gel for removal of textile dyes and other pollutants from aqueous media are listed in the Electronic Supplementary Information (ESI 2) (Kabir et al., 2013; Tabesh et al., 2018). Sol-gel preparation includes hydrolysis of suitable precursors, usually metal alkoxides M (OR)x, and their subsequent condensation, with the release of either water or an alcohol (Oubaha, 2019). Thus, the reaction involves substitution of alkoxyl groups with hydroxyl groups, followed by condensation of partially hydrolyzed alkoxides (Rocío-Bautista et al., 2016; Oubaha, 2019). The most common and extensively used Sol-gel techniques as solid extractors involve the use of α-Al_2_O_3_ nanoparticle–modified Sol-gel (Tabesh et al., 2018), metal-organic frameworks (Jalili et al., 2020), and dispersive solid-phase microextraction techniques (El-Shahawi et al., 2005a; Ghorbani et al., 2019; Murtada, 2020).

The choice of the target pollutant EY was due to its wide applications in industries and its subsequent discharge as an effluent as well as its toxic impacts on retinal ganglion cells, inhalation toxicity, and lifetime toxicity/carcinogenicity (Baharak and Ali, 2013; Bassey et al., 2012; Pavlidis et al., 2003; Sun et al., 1987). To the best of our knowledge, no studies on the use of the Sol-gel modified PUFs as solid phase microextractors for textile dyes removal have been published to date. Understanding the processes that control the behaviour of the Sol gel/PUFs extractor toward EY sorption at aqueous media interfaces also permits its use as a proxy for the reconstruction of new extractors for dyes, even at trace levels in water, and allows investigation of the role of Sol gel immobilized PUFs.

With this background in mind and taking into account the key characteristics of the PUFs and the crucial features of the Sol gel, the combination of Sol gel and PUF materials could be an ideal, perfect, and eco-friendly approach to establish a new d-µ-SPME for wastewater remediation and precise monitoring of EY dye. Therefore, the overall aims of the current study were focused on: i) the fabrication and characterization of the Sol gel and the Sol gel impregnated PUFs sorbent; ii) studying the impact of various parameters that facilitate EY dye retention from the aqueous media by the Sol-gel modified PUFS; iii) assigning the kinetics and thermodynamics of the EY dye sorption from aqueous media, and finally iv) testing the re-usability of the established Sol-gel dispersed PUFs extractor. Sol co-compositing may also improve the sorption characteristics, which cannot be attained by either of the components alone. The current strategy will also contribute effectively to further refining the removal and/or minimization of dye residues from the aquatic environment, e.g., industrial wastewater and underground water samples. A cohesive collaboration between industry and academic institutes will be anticipated to allow market distribution and mass fabrication of Sol-gel/PUFs platforms.

## 2 Experimental

### 2.1 Reagents and materials

White sheets of commercial polyether-type based polyurethane foams (PUFs) were purchased from the local market of Jeddah City, Saudi Arabia. Analytical reagent (AR) grade chemicals were used as received. PUF cubes approximately 1 cm^3^ were cut from the PUF sheets and were washed with HCl (10% v/v) followed by deionized water until the washing solutions were free from chloride ions (El-Shahawi, 1994; El-Shahawi and Alwael, 2019). The PUF cubes were then washed with acetone to remove any organic contaminates or monomers, dried for 2 h in an oven at 80°C, and finally stored in dark, clean bottles for use (El-Shahawi et al., 2005a). A standard sock solution (1.0 mg mL^−1^) of EY dye was prepared in deionized water (100 mL) and diluted working solutions (0.001–70 μg mL^−1^) of the EY dye were prepared by appropriate dilution of the stock solution. A series of Britton-Robinson (B-R) buffer solutions of pH 1.8–12 were prepared, as reported earlier (Vogel et al., 1952). The impact of acidity of the extraction media was studied using HCl (0.01–1 M).

### 2.2 Instrumentations

The particle sizes of the synthesized Sol-gel were determined using a Malvern Nano-ZS dynamic light scattering (DLS) instrument. To prevent aggregation of the particles and to enable DLS characterization of the individual particles, the Sol-gel was initially filtered through 0.45 μm Whatman syringe filters and diluted with isopropyl alcohol to a 1:10 (v/v) ratio. A scanning electron microscope (SEM) (JEOL-JSM6301-F) (Peabody, MA, United States) coupled with an ALS2300C model energy dispersive spectroscopy (EDS) analyser (Germany) using an accelerating voltage of 5 keV for SEM images and 20 keV for EDX analyses was used for characterization of the surface topography and roughness of the Sol gel and Sol gel impregnated PUFs. Prior to analysis, the samples were mounted using a mixture of Epo Fix resin and Epo Fix hardener (w/w 20:3). The mounted samples were polished using different grades of emery paper (from coarse to fine), namely, P240, P500, P800, and P1200. The final polish was carried out using a diamond suspension with particle sizes of 9, 3, and 1 µm. The polishing process was carried out using a Motopol Tm 2,000 grinder/polisher. The polished samples were coated with approximately 4 nm of platinum/palladium using a Cressington 208HR sputter coater to make the sample conductive and to minimize charging during image recording.

A Perkin Elmer GX FTIR instrument (4,000–650 cm^−1^) was used for recording FTIR spectra in the reflection ATR configuration for the Sol gel and Sol gel impregnated PUFs in order to allocate the different vibrational modes of the functional groups of the Sol gel and PUFs and to assign the impact of the functional silanes on the structure of the reference sol-gel material. FTIR spectra of the Sol-gel and Sol gel treated PUFs were recorded on a thin coating deposited on a glass slide and dried at 100°C for 1 h to eliminate any solvent. A UV-Vis spectrophotometer (Shimadzu UV-Vis 1800, Japan) (190–1,100 nm) was used for recording the electronic spectra and absorbance data of the EY dye before and after extraction by the Sol gel treated PUFs. A Corporation Precision Scientific mechanical shaker (Chicago, CH, United States) with a shaking rate in the range 10–250 rpm and a thermostatically controlled shaker (GFL-1083 model, Germany) were used in batch experiments. A pH meter (Model inoLab™ Multi 9,430) and a Milli-Q Plus system (Millipore, Bedford, MA, USA) were used for pH measurements and for providing ultra-pure water, respectively. A series of digital micropipettes (Volac) (Model 3,505, UK) was used for the preparation of more diluted EY solutions.

### 2.3 Recommended methodology

#### 2.3.1 Preparation of the Sol-gel nanomaterials and binding to PUFs

The newly prepared hybrid sol-gel surface treatment consisted of preparing a functional nanoparticle-based sol that would enable the preparation of sub-micron thin coatings, called surface treatments. The employed methodology consists of enabling the formation of sensitive amino-functional groups at the surface of the foam material. To do this, the principle consisted of employing a sol-gel reactive aminosilane precursor, namely, 3-methacryloxypropyl-trimethoxy -silane (MAPTMS) and growing nanoparticles through hydrolysis and condensation sol-gel reactions that would be adequately dispersed in a solvent. Thus, the Sol gel matrix was prepared via hydrolysis and condensation reactions of the silicon precursor MAPTMS (99% in methanol, Aldrich) and the modified organic zirconium complex, prepared from the chelation of Zr^4+^-*n*-propoxide (ZPO) (assay 70% in propanol, Sigma Aldrich, Irl.), at a ratio of 80:20, with the theoretical hydrolysis degree being 50% against the total content of reactive alkoxide groups. The synthesis required a three-step process, as demonstrated in ESI 3 as follows (Cullen et al., 2017; Oubaha, 2019): i) pre-hydrolysis of the MAPTMS and complexation of ZPO with the chelating agent, ii) addition of the pre-hydrolysed alkoxysilane within the zirconate complex, and finally iii) hydrolysis of the solution mixture, where MAPTMS is pre-hydrolysed using an aqueous HNO_3_ (0.1 M) solution with a 1:0.25 ratio. The hydrolysis is performed in a heterogeneous way within 5 min until methanol production becomes sufficient enough to enable miscibility of all species in solution. In parallel, ZPO is chelated with MAAH to block two of its alkoxide groups and minimize precipitation when it is in contact with water. These two reactions are achieved simultaneously, and the solution was allowed to stir for 45 min and the partially hydrolysed MAPTMS was added gradually drop wise to the Zr (VI)-complex. Neutral hydrolysis was performed after 5 min with deionised water by drop wise addition to the mixture. A diagram describing the Sol gel preparation is shown in ESI 3. Aggregation of the particles during the Sol-gel reaction process was minimized by adding isopropyl alcohol (IPA) solvent and enabling the formation of small particle sizes. IPA solvent can simultaneously enable dispersion of the particles while acting as a hydrophilic medium and facilitating adhesion of the particles to the PUF surfaces during impregnation of the Sol gel onto the PUF surface. Additionally, once the adhesion of the particles has taken place, IPA can easily evaporate, thus leaving only the reactive species at the PUF surface.

#### 2.3.2 Synthesis of Sol-gel functionalized polyurethane foams (d-µ SPME)

An accurate mass (1.0 g) of the PUF cubes was dried and shaken, with the Sol-gel dissolved in isopropanol (IPA) (4% v/v) with constant stirring for 30 min. After shaking, the Sol-gel impregnated PUF cubes were then separated out and dried between sheets of filter paper as, described earlier (El-Shahawi et al., 2005ba; Han et al., 2017). Binding of the PUF surface with the Sol gel (silanol species) is possible via the occurrence of covalent or strong H-bonding with the PUF surface. IPA enables dispersion of the particles and facilitates the adhesion of the particles to the PUF surface during the impregnation process. Moreover, once the adhesion of the particles has taken place, IPA can easily evaporate, thus leaving only the reactive species at the surface of the substrate.

#### 2.3.3 Batch (static) experiments

Accurate masses (0.1 ± 0.002 g) of the Sol-gel functionalized PUFs in the conical flasks (100 mL) were shaken with a standard concentration of EY (10 μg mL^−1^) at pH 3.0 for 60 min on a mechanical shaker at room temperature (25°C ± 1°C). The test aqueous solutions were separated out by decantation after equilibration, and the concentration of the EY dye remaining in the aqueous phase was determined by measuring the absorbance of the dye solution at 517 nm (λ_max_) *versus* the reagent blank. The EY dye retained on the Sol-gel treated PUFs was subsequently calculated from the difference (A_b_−A_f_) between the absorbance of the dye in the aqueous phase before (A_b_) and after extraction (A_f_). The extraction percentage (%E), partition ratio (D), and amount of EY dye retained at equilibrium (q_e_, mg/g) at a time t (q_t_, mg/g) by the used Sol-gel–treated PUFs extractor were calculated, as reported earlier (Amiri, 2016; Kabir et al., 2016).

#### 2.3.4 Validation and analytical utility *of the established extractor*

Environmental marine water samples collected from the north coast of Jeddah, KSA were primarily filtered through a membrane filter (0.45 µm). The pH of the water samples was then adjusted to pH 4 using a Britton Robinson buffer and the samples were spiked with known concentrations (5–30 μg mL^−1^) of the EY dye. The water samples were subsequently passed through Sol gel/PUF sorbent-packed mini columns at reasonable flow rates (3–5 mL min^−1^) *versus* the reagent blank. Complete EY retention was achieved, as confirmed from the absorbance of the dye in the effluent solution at 517 nm (λ_max_) *versus* the reagent blank. The retained EY species were recovered with NaOH (5 mL, 0.1 M) at a flow rate of 2.0 mL min^−1^.

## 3 Results and discussion

### 3.1 Preliminary study

Recently, the selection and/or development of an effective and low-cost dispersive solid phase microextractor (d-µ SPME) for water treatment has become vital (Ghorbani et al., 2019; Murtada, 2020). The strong interaction of the target analyte in the aquatic environmental samples with the established solid extractor and its available active sites are also important. Thus, the establishment of an extremely efficient, high sorption capacity, dispersive solid phase microextractor sorbent for complete removal of the target textile dye from the aqueous phase is of prime importance (Ghorbani et al., 2019). PUF solid sorbents are an example of one of most important three-dimensional (3D) products available for fabricating super hydrophobic absorbents for many organic and inorganic complex species (Han et al., 2017; El-Shahawi and Alwael, 2019). PUF sorbents have received great attention because of their excellent hydrophobicity and oleophilicity characteristics, which are required for solid phase absorbents used for various polar and non-polar species, as well as their hydrophobicity, low cost, availability, and simplicity of preparation (Amiri, 2016; Han et al., 2017). PUFs also have great permeability, convection dominated mass–transfer, a unique membrane-like structure, resilience properties, and are easy to modify and chemically stabilize at a wide range of pHs compared to other conventional sorbents (Gunashekar and Abu-Zahra, 2015; Kochane et al., 2017). Thus, great attention has been oriented toward the modification of PUFs with nanomaterials.

The choice of nanomaterials with outstanding properties, such as small particle size and high surface area, e.g., Au nanoparticles, Sol gel etc., for PUF modifications makes PUFs an ideal model platform toward the complete removal of the potent target species (Kochane et al., 2017; El-Shahawi and Alwael, 2019). Preliminary studies on the extraction of EY dye from an aqueous solution with pH < 3 using the developed Sol-gel functionalized PUFs sorbent revealed a considerable uptake (>90%) of the EY dye compared to untreated PUFs (20%–25%) (ESI 4). Thus, in the sequence study, characterization, retention profile, and the analytical utility of the Sol-gel treated PUFs as a d-µ SPME platform for the complete removal, recovery, and subsequent spectrophotometric ET determination, are discussed below.

### 3.2 Characterization of Sol-gel

**FIGURE 1:**
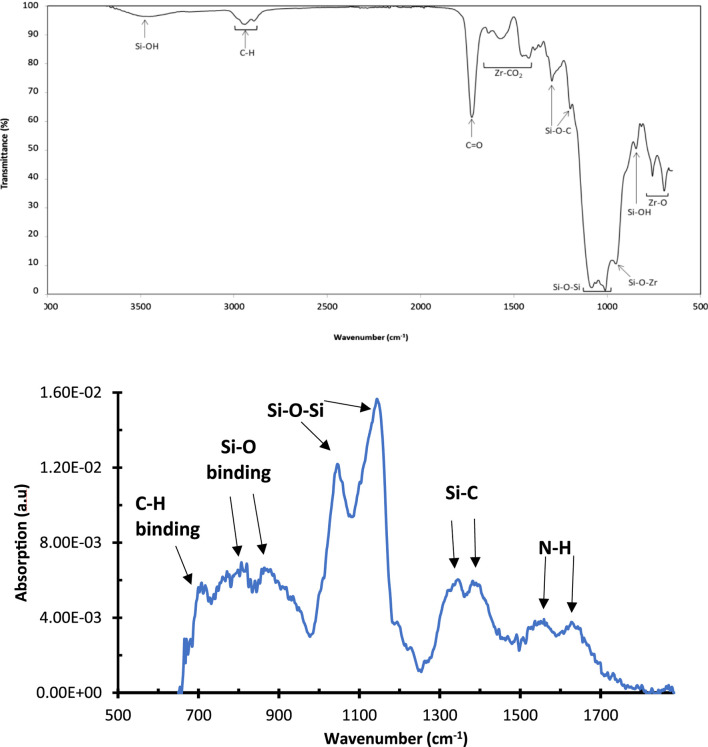
FTIR spectrum of the Sol-gel in the range 500–4,000 cm^−1^.

FTIR of the synthesized Sol gel was recorded on a thin coating deposited on a glass slide and dried at 100°C for the complete removal of all solvents. In the FTIR spectrum (Figure 1), the vibration located at 840 cm^−1^ was assigned to the presence of residual uncondensed silanol species (Si-OH) (Cullen et al., 2017; Oubaha, 2019) and the vibrations located at 950 and in the range 1,000–1,100 cm^−1^ were assigned to condensed silicon species, including silicon/zirconium oxide (Si-O-Zr) and siloxane (Si-O-Si) groups, respectively, as reported earlier by Oubaha et al. (Cullen et al., 2017; Oubaha, 2019).

The vibrations at 1,175 and 1,295 cm^−1^ were assigned to Si-O-C groups of the residual methoxysilane moieties and the vibration band at 1725 cm^−1^ was assigned to the C=O contained in the MAPTMS precursor. The band centred at 3,450 cm^−1^ is typical of Si-OH vibrations (Nakamoto, 1986; Oubaha, 2019). The bands located at 1,400–1,600 cm^−1^ were attributed to the Zr-CO_2_ groups contained in the zirconium complex, arising from the chelation of zirconium propoxide and methacrylic acid (Cullen et al., 2017; Oubaha, 2019). These results suggested that the formation of condensed silicon and zirconium species has taken place and confirms the occurrence of Sol-gel hydrolysis and condensation reactions between the inorganic silicon and zirconium alkoxide groups (Cullen et al., 2017; Oubaha, 2019).

**FIGURE 2:**
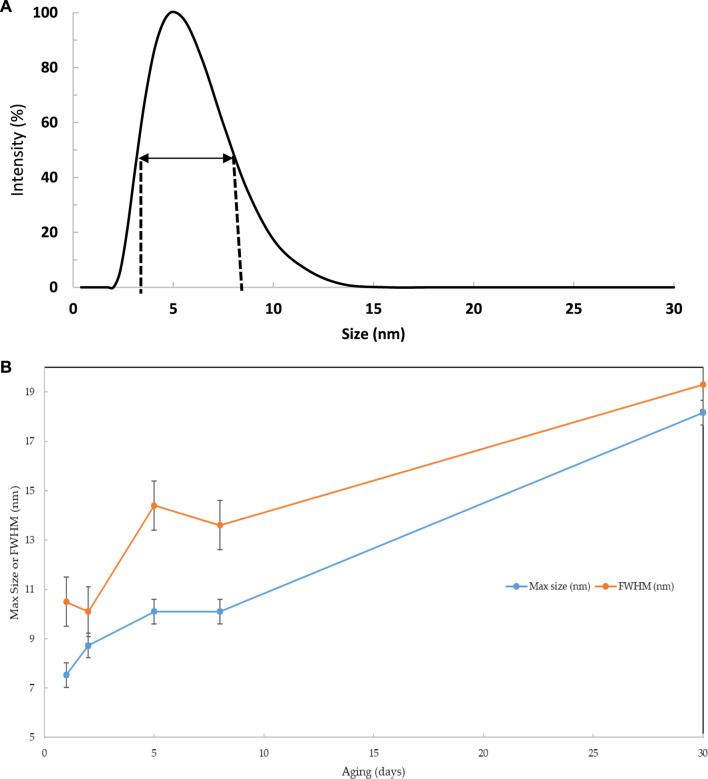
DLS analysis of the hybrid Sol-gel materials over 30 days ageing **(A)** and the full width at half maximum (FWHM) and maximum peak position of the reference material over 30 days **(B)**.

The functionality aspect of the Sol-gel and its morphology, including its particle size and surface area, may play an essential role in the accessibility of the tested textile dyes in the test aqueous solution. Thus, DLS analyses 1, 2, 5, 8, and 30 days after the preparation of the Sol-gel material were recorded, and representative results are demonstrated in Figure 2A. DLS analysis was critically used to determine the particle size and homogeneity of the prepared Sol-gel prior to impregnation onto the pre-washed PUFs and its evolution with time (Cullen et al., 2017; Oubaha, 2019). This feature is important to identify the possible morphology of the Sol-gel material in respect to the subsequent extraction performance of the proposed solid platform. The first observation is the appearance of one band in the range 2–50 nm, centred between 5–20 nm, indicating that the network is composed of a distribution of particles, with most ranging between 5 and 20 nm (Cullen et al., 2017; Oubaha, 2019). Very small particle growth within the Sol-gel over 30 days was also observed, and the Sol gel reached its maximum peak at 8 days.

The full width at the half maximum (FWHM) of the observed bands was calculated to determine the dispersion and populations of the nanoparticle. The data are shown along with the FWHM in Figure 2B. The bands are shifted toward higher sizes, where the DLS of samples aged for 1–5 days showed a single band for samples with aging from 8 to 30 days. The Sol-gel particle size increased quite rapidly from 7.5 to 10 nm within the first 5 days and increased from 10 to 18 nm until 30 days of aging. The appearance of small particles in conjunction with the shift of the large band toward higher sizes above 5 days of aging revealed that the growth process is mainly linked to the aggregation of small particles on the surface of larger particles during the first 5 days of aging. The formation of small particles above this age is favoured, probably due to the presence of less reactive groups at the surface of the larger particles. The progressive increase of the FWHM within the studied period suggests a decrease in the homogeneity of the Sol-gel system.

**FIGURE 3:**
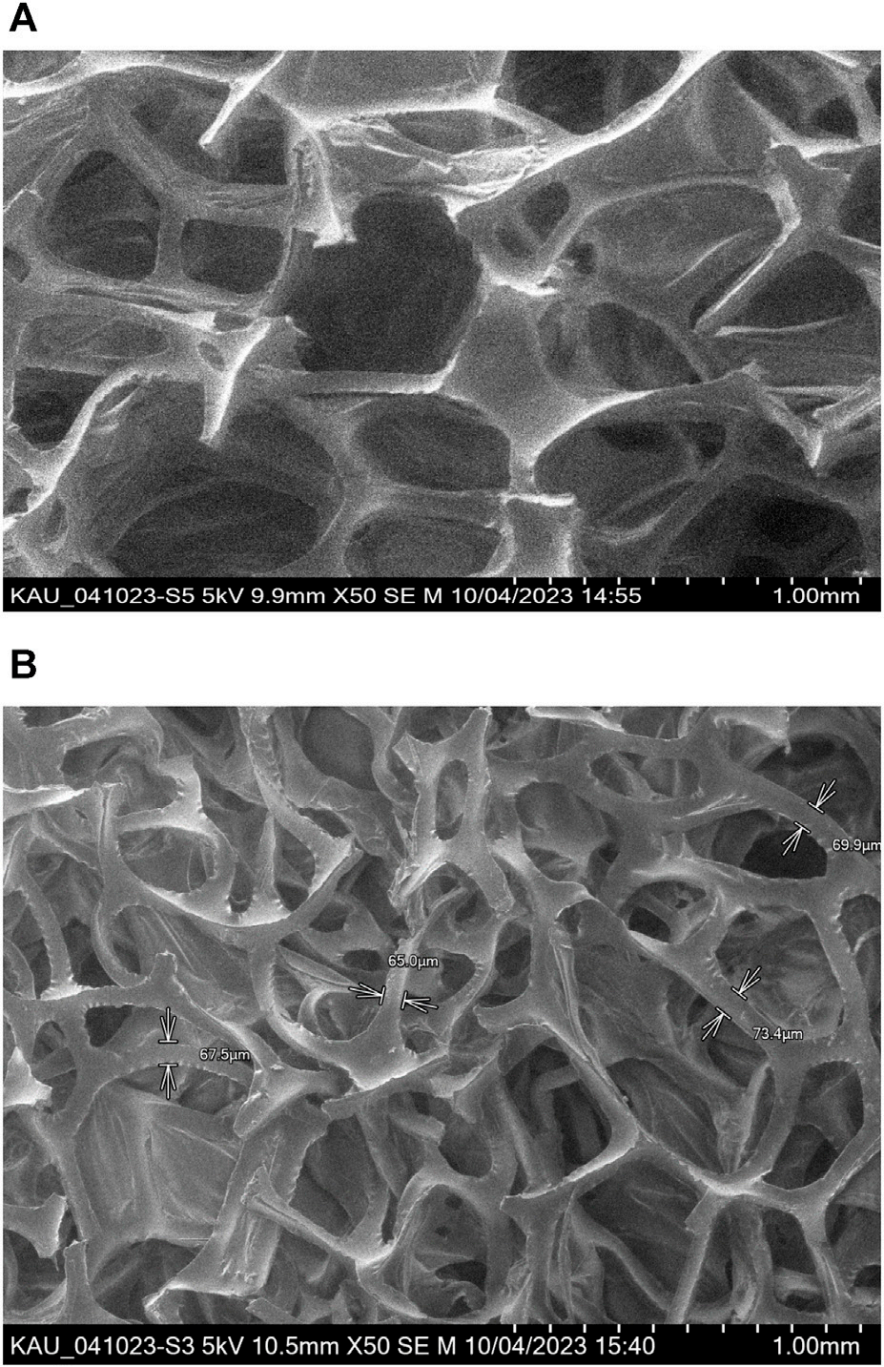
SEM images of PUFs **(A)** and Sol gel–treated PUFs structure **(B)**. Top 9.8 × 50; Bottom-10.5 × 50.

SEM images of the PUFs and Sol-gel treated PUFs are shown in Figures 3A, B. The SEM images displayed the membrane and the polyhefral-like structure of the PUFs with and without Sol-gel (Han et al., 2017). The SEM image of the Sol-gel/PUFs (Figure 3B) clearly shows the presence of a uniform and continuous coating with a thickness of 159 ± 0.2 nm, homogenously deposited at the surface of the PUFs material. The polyhedral, on average, is clearly visible as a quazi-spherical pentagonal dodecahedra, in close agreement with reported data (Han et al., 2017). Moreover, the polymer and Sol gel are homogeneously distributed between the walls of the bubbles and the lines where bubbles intersect.

**FIGURE 4:**
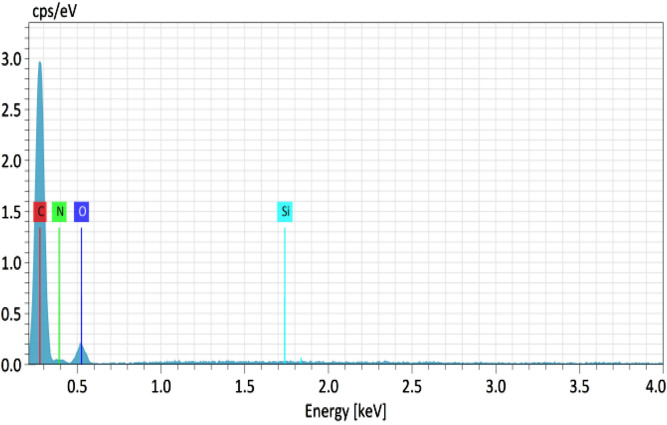
EDX spectrum of the Sol gel–immobilized polyurethane foams.

The chemical composition of the Sol gel and Sol gel/PUFs was further supported by recording their EDS spectra. A representative EDS spectrum of the Sol gel/PUFs is shown in Figure 4. The EDS spectrum of the Sol gel/PUFs displayed peaks at 0.09, 0.25, 0.5, and 1.75 keV, revealing the presence of C, N, O, and Si elements in the composite, with average mass concentrations (%) of 68.82, 4.79, 23.27, and 3.13, respectively. Moreover, the EDS spectrum of the PUFs displayed well defined peaks at 0.09, 0.25, and 0.5 keV, close-fitting to C, N, and O, with average mass concentrations (%) of 80.35, 2.82, and 16. 75, respectively. These data confirmed the impregnation of the Sol-gel material into PUFs. The Sol gel can be classified as a nanomaterial that can be immobilized onto PUF sorbents, and it will be tested at times not greater than 1 day of aging to maximize its surface area and surface reactivity.

### 3.3 Sorption characteristics of EY dye onto the developed sol-gel/PUFs

A preliminary study on the extraction of EY dye from the aqueous solution with pH < 3 by the established Sol-gel–functionalized PUFs sorbent revealed a considerable uptake (>90%) of the EY dye onto the Sol-gel–treated PUFs sorbent compared to the dye uptake onto untreated PUFs (10%–15%) (ESI 4). Thus, in the sequence study, Sol-gel treated PUF sorbents were successfully used as a d-µ SPME for dye removal and subsequent recovery from the test aqueous media. The nanosized Sol-gel provides a high surface area, H-bonding through Si-O-Zr and siloxane (Si-O-Si) groups of the Sol gel, and specific sorption affinity toward the analyte after impregnation on an extractor such as PUFs (Kabir et al., 2013; Amiri, 2016). The PUFs membrane-like structure, in addition to H-bonding, including surface complexation and electrostatic π-π interaction resulting from the dye and silicon/zirconium oxide (Si-O-Zr) and siloxane (Si-O-Si) groups on the sorbent, facilitate its analytical utility as an excellent and resilient solid platform sorbent material for complete sorption of dye species from aqueous media. PUFs sorbent also have numerous advantages, e.g., commercially available, low cost, and easy to prepare and handle compared to most common solid extractors (Han et al., 2017; El-Shahawi and Alwael, 2019). PUFs have a distinctive feature as an easy to modify solid sorbent and are different from other solid sorbents (Gunashekar and Abu-Zahra, 2015; Han et al., 2017; Kochane et al., 2017). Moreover, the volume-to-surface ratios of the quasi-spherical membrane geometry, in addition to the cylindrical and/or planar membrane geometry, are the main characteristics of PUFs. Thus, the utility of the Sol-gel/PUFs sorbent as a dispersive microextractor platform for complete removal, recovery, and subsequent dye determination, are discussed below.

Recently, nanoparticle technology has played a key role in providing opportunities and possibilities for the development of a new generation of sensing and preconcentration tools. Thus, the targeted solid phase microextraction-based Sol-gel technique has become a major research thrust in the last few years (Kabir et al., 2013; Tabesh et al., 2018) because of its low cost and effective and greener dispersive solid phase micro extractors (d-µ SPME) that are eco-friendly and reliable for the complete removal and subsequent trace determination of a series of inorganic and organic species, including textile dyes, from the aquatic environment (Jalili et al., 2020; Murtada, 2020).

**FIGURE 5:**
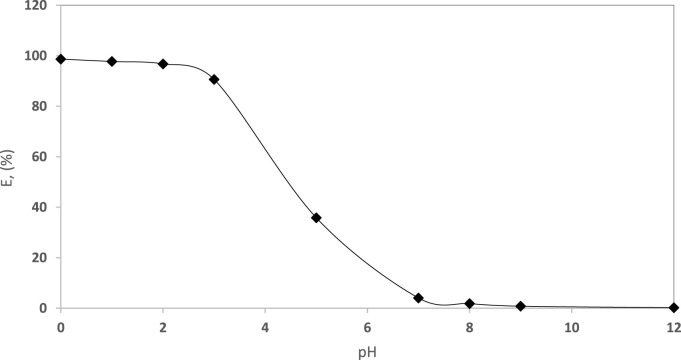
Influence of pH (pH 0.0–12.0) of the aqueous solution for EY (10 μg mL^−1^) dye uptake by the established sol-gel/PUFs (0.1 ± 0.002 g) after 60 min shaking at 25°C ± 1°C.

The impact of the solution pH (pH 0.0–12.0) on the EY dye retention by the established Sol-gel/PUFs was studied after 60 min of shaking at room temperature, using batch mode of separation. The results are shown in Figure 5, where maximum dye retention was achieved with a pH (after extraction) in the range of 0.0–3.0. The extraction percentage of EY decreased at pH≥ 3.8 (Figure 5), in good agreement with the results reported by Kochane et al. (Kochane et al., 2017) and Abu-Zahra and co-workers (Farag et al., 2007; Hussein and Abu-Zahra, 2016; Hussein and Abu-Zahra, 2017; Al-Bagawi et al., 2018). At pH <3.8, the Sol gel/PUFs sorbent linkages are present in the protonated ether form (–CH_2_–OH^+^– CH_2_–) and/or the urethane form (–NH_2_^+^– COO–), as reported earlier (Farag et al., 2007; Al-Bagawi et al., 2018). At pH ≤ 3.8, the possibility of forming binary complex ion associates with the dye anion via the ether (-O-) and/or urethane (-NH-CO-)- groups of the PUFs of the Sol-gel/PUFs extractor, in addition to the electrostatic attraction, are high, resulting in an increase in dye retention, as previously reported (Farag et al., 2007; Hussein and Abu-Zahra, 2017). Based on the theory of electrostatic interaction (Ghorbani et al., 2019; Jalili et al., 2020; Murtada, 2020), the presence of the dye anion encourages the formation of a binary ion associate with the protonated ether and/or urethane linkages on/in modified d-µ SPME PUFs sorbent membranes at pH ˂ 3.8. Thus, the behaviour can be represented as follows:

i) Based on the ether oxygen group of PUFs (Eqs 1, 2):

$${\left(-{\text{CH}}_{2}-O-{\text{CH}}_{2}-\right)}_{\text{PUFs}}+H^{+}⇌{\left(-{\text{CH}}_{2}-{\text{HO}}^{+}-CH_{2}-\right)}_{PUFs}$$ (1)

−CH2 −HO +−CH2‐ PUFs+Dye anionaq‐ ⇌ ‐CH2‐HO+‐CH2. Dye anion‐PUF (2)

ii) Based on PUFs urethane N (Eqs 3, 4) and Sol-gel/PUFs (Eq. 5):

$${\left(-\text{NH}-\text{COO}-\right)}_{PUFs}+H^{+}⇌{\left({\text{NH}}_{2}^{+}-\text{COO}-\right)}_{PUFs}$$ (3)

$${\left(-{\text{NH}}_{2}^{+}-\text{COO}-\right)}_{PUFs}+{\left(\text{Dye}\;\text{anion}\right)}_{aq}^{-}⇌{\left\{\left[-{\text{NH}}_{2}^{+}-\text{COO}-\right].\;{\left[\text{Dye}\;\text{anion}\right]}^{-}\right\}}_{PUFs}$$ (4)

Sol‐gel+PUFsDye anionaq‐ ⇌ Sol‐gel.dye anion‐PUFs (5)

The possibility of forming H-bonding between the complex ion associate of the Sol-gel/PUFs and the dye anion, the strong electrostatic interaction between the bulk dye anion and the PUFs, and the large available effective surface area of the Sol-gel/PUFs (d-µ SPME) are the most likely cause of dye retention at a lower pH and may account for the trend observed. At pH > pH 3.8, the dye retention was low and almost reached zero extraction percentage at pH ≥ 7, which is most likely attributed to deprotonation of the protonated ether and/or urethane of the PUFs, as well as the existence of the EY dye in anionic form at this pH (pH >7) in the aqueous media and the weak electrostatic interaction between the Sol-gel–modified PUFs (d-µ SPME) sorbent membranes and the dye, resulting in minimal dye retention on/in the sorbent (Ghorbani et al., 2019; Jalili et al., 2020).

### 3.4 Sorption kinetics

Transport of solute analyte molecules from the aqueous solution to the surface of the solid phase extractor, followed by the passage of molecules into the interior of the solid pores and membranes are the utmost collective steps in separation by solid phase extraction. The EY retention sorption from the aqueous solution at the optimized pH (at pH ˂ 3.8) onto the Sol gel/PUFs sorbent was found to depend on the time (5–60 min) of shaking and the dye retention was found to be fast and reached equilibrium within ∼60 min of the shaking period (ESI 5). The dye retention was fast at the early stage of the shaking period, where over 60% of the EY dye was sorbed within 5 min of shaking and the dye uptake was found to be slow, with equilibrium achieved after 30 min, where more than 95% of the EY dye was retained. This assumption was supported by the value of the half-life time (t_1/2_) (t_1/2_ = 0.8 ± 0.1 min) of the dye sorption, illustrating the good performance of the established Sol gel–modified PUFs extractor for EY dye retention from water (ESI 6). To identify the key process controlling the EY dye retention, several models must be checked for suitability and consistency over a broad range of system parameters. Thus, the data were subjected to different kinetics models, e.g., diffusion-based Weber–Morris (Weber and Morris, 1964), Lagergren (pseudo-first order) (Lima et al., 2015), and pseudo-second order (Ys and McKay, 1999).

Initially, to reveal the relative contribution of surface and intraparticle diffusion, the sorption data were exposed to the Weber–Morris model (Weber and Morris, 1964) to determine whether intraparticle diffusion or film diffusion is the rate controlling step of EY sorption by the Sol gel/PUF sorbent. This model can be stated by the following Eq. (6):

$$q_{t}=R_{d}\sqrt{t}+C$$ (6)

where, R_d_ and q_t_ are the rate constant of intraparticle transport (mg g^−1^ min^−1/2^) and equivalent quantity of retained analyte (mg g^−1^) at a time t, respectively, and the term C (mg g^−1^) represents the value of the intercept from the initial linear portion of the linear plot associated with the thickness of the boundary layer, where a higher C value corresponds to a greater effect of the boundary layer (Weber and Morris, 1964). The plot of q_t_ *versus* the square root of time ( $\sqrt{t}$ ) was linear at the initial stage and deviated when the shaking time increased (ESI 7). The R_d_ values, as computed from the two slopes in the initial and second stages of the Weber–Morris plot (ESI 7) for EY retention by the established sorbent, were found to equal 1.893 and 0.175 mg g^−1^ min^−1/2^, with correlation coefficients (R^2^) of 0.985 and 0.992, respectively. The change of the pore volumes and the exhaustion of the Sol gel PUFs sorbent are most likely attributed to the change of the slopes. The plot did not pass through (C = 0.0); therefore the adsorption kinetics of EY onto the sorbent is regulated by both surface and intraparticle diffusion processes.

The low value of the constant C (0.244 mg g^−1^) shows that film diffusion plays less of a role in the rate-controlling step and that the intraparticle diffusion step can be considered as the rate-controlling step (Olu-owolabi et al., 2014). Thus, it can be concluded that the first stage occurred instantly and rapidly after transferring the dye from the bulk solution, i.e., “bulk transport” is more predominant. The second stage involves transportation of the sorbate molecules from the bulk liquid phase to the external surface of the sorbent, through a hydrodynamic boundary layer or film solution (known as “film diffusion”) and, finally, diffusion of the sorbate molecules from the exterior of the sorbent, i.e., “external diffusion” (Olu-owolabi et al., 2014). Thus, the rate of EY dye sorption at the early stage of extraction is controlled by film diffusion (Weber and Morris, 1964; Olu-owolabi et al., 2014), whereas intraparticle diffusion of the dye and the fact that dye sorption decreased in the second stage indicate that exhaustion of the pore volume of the sorbent is most prevalent (Ys and McKay, 1999; Olu-owolabi et al., 2014).

The data were also subjected to a pseudo–first order (Lagergren) model (Lima et al., 2015) using Eq. (7):

$$\log\left(q_{e}-q_{t}\right)=\log q_{e}-\;\frac{k_{1}}{2.303}t$$ (7)

where, k_1_ (min^−1^) is the first order rate constant of dye sorption per minute and q_e_ and q_t_ (mg g^−1^) are the quantities of sorbed dye per unit mass of the established solid extractor at equilibrium (q_e_) and at a time t (q_t_), respectively. The plot of log (q_e_–q_t_) *versus* time was linear (ESI 8), with a correlation coefficient of R^2^ = 0.9961. The calculated value of the first order rate constant k_1_, as calculated from the slope of the linear plot, was found to be 0.081 min^−1^. The good agreement between the experimental value of q_e_ (19.75 mg g^−1^) and the computed value of q_e_, (18.92 mg g^−1^) is most likely ascribed to the occurrence of a boundary layer or external resistance governing the uptake step at the primary stage of the retention process (Lima et al., 2015).

**FIGURE 6:**
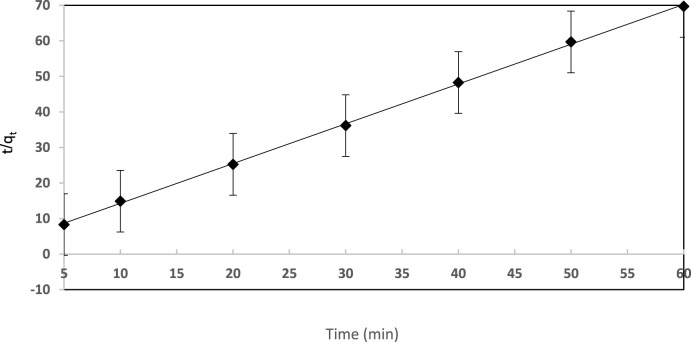
Pseudo-second order kinetic plot for EY (10 μg mL^−1^) retention on the sol-gel/PUFs (0.1 ± 0.002 g) solid extractor at pH 3.0 and 25 ± 1°C.

The data for EY dye uptake was further exposed to a pseudo-second-order model (Eq. 8) (Ys and McKay, 1999; Olu-owolabi et al., 2014):

$$\frac{t}{q_{t}}=\frac{1}{h}+\left(\frac{1}{q_{e}}\right)t$$ (8)

where h = k_2_q_e_^2^ can be assumed as the initial rate constant of the sorption step and k_2_ is the pseudo-second order rate constant g (mg^−1^.min^−1^). q_e_ and q_t_ (mg g^−1^) refer to the quantities of sorbed dye per mass of sorbent at equilibrium and at any time t (min), respectively. The plot of t/q_t_ *vs*. time for dye sorption by the established solid extractor was found to be linear (Figure 6). According to the correlation coefficients, the pseudo-second-order equation (R^2^ = 0.999) more accurately describes the data than the pseudo-first-order equation (R^2^ = 0.995). The q_e_ value (36.44 mg g^−1^) calculated from the pseudo-second order model is quite close to the q_e_ value (37.64 mg g^−1^) evaluated from the experimental batch experiment. These outcomes confirmed the fitness of the pseudo-second order rate equation for the description of dye sorption by the established sorbent. Please insert Figure 6.

### 3.5 Thermodynamic characteristics

**FIGURE 7:**
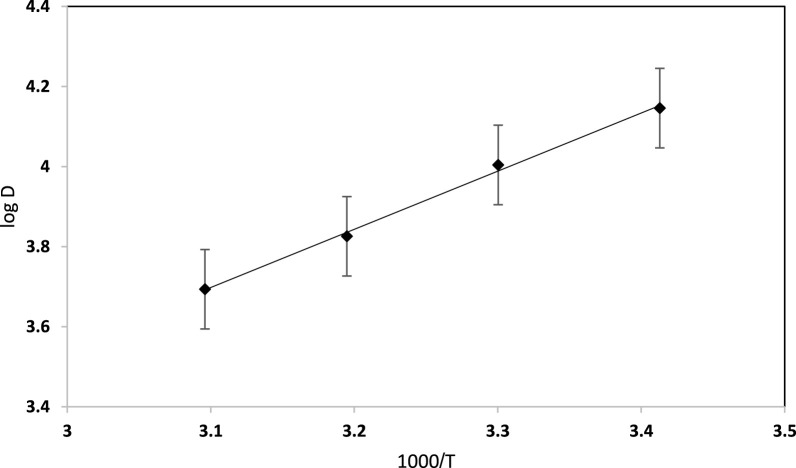
Vant-Hoff plot for EY (10 μg mL^−1^) dye sorption from aqueous solution onto sol-gel/PUFs (0.1 ± 0.002 g) at 293 K.

Temperature is a crucial parameter influencing dye retention, where the solution viscosity tends to decrease with increasing temperature, resulting in more effective diffusion rates of the sorbent, which eventually modify the sorption capacity. Thus, the impact of temperature on dye uptake by the used solid platform at the optimal analytical parameters of dye retention was studied over a wide range of temperatures (293–323 K). Sorption thermodynamic studies, providing Gibbs free energy (ΔG^⁰^), entropy change (ΔS^⁰^), and enthalpy change (ΔH^⁰^), are an integral part of the study and can be useful to explore how the removal mechanism (i.e., physisorption and chemisorption) is affected by temperature changes. The plot of the distribution ratio (log D) *versus* 1,000/T was linear, as shown in Figure 7. The dye retention increased when the temperature decreased, supporting the exothermic nature of EY dye uptake by the Sol gel–modified PUFS extractor. The numerical values of the thermodynamic parameters ΔH^⁰^, ΔS^⁰^, and ΔG^⁰^, as calculated from the slope and intercept of the linear plot of log D *versus* 1,000/T (Lima et al., 2015), were found to equal −69.58 kJ mol^−1^, 147.65 J mol^−1^ K ^−1^, and −78.07 kJ mol^−1^ (at 293 K), respectively. The negative value of ΔH^⁰^ (−69.58 kJ mol^−1^) indicated that the reaction was exothermic. Further, the positive value of ΔS^⁰^ (147.65 J mol^−1^ K^−1^) revealed that the dye retention most likely increased the randomness at the solid-liquid interfaces during the sorption step (Hu et al., 2013; Olu-owolabi et al., 2014; Ahmed et al., 2021). Further, the decrease of ΔG^⁰^ values clarifies that dye sorption is favoured at elevated temperature on the established sorbent (Lingamdinne et al., 2017). The mobility of the textile dye increases upon raising the temperature, causing escape of the analyte molecules from the solid phase to enter the liquid phase. The ΔH value can provide evidence of the type of interaction (s) associated with the sorption process. The ΔH value was well-suited with energy strengths associated with weak hydrophobic forces, e.g., Van-der Waals and electrostatic π-π interactions, which are common for hydrophobic compounds with little or no polarity (Olu-owolabi et al., 2014; Veerakumar et al., 2017).

### 3.6 Possible retention mechanism

Understanding the mechanism of dye retention by the developed extractor, even at low levels in aqueous media interfaces, permits its use as a substitute for the re-establishment of a new extractor for dyes in water. Taking into consideration the impact of the analytical parameters, the sorption isotherm models of EY sorption by the Sol-gel/PUFs, the key characteristics of the PUFs and Sol gel, and the combination of both materials as an ideal and eco-friendly solid sorbent for wastewater remediation, it can be proposed that the dye retention is most likely attributed to “weak base anion exchanger of” {[–CH_2_–OH^+^ –CH_2_–]. [Dye anion]^−^ }_Sol-gel_ _PUF_ and/or {[–NH_2_^+^ –COO–]. [Dye anion]^−^ }_Sol-gel_ _PUF_ via “solvent extraction,” in addition to the “surface adsorption” of the formed complex ion associate of the PUFs and the dye anion on/in the Sol-gel/PUFs membranes. Hence, the overall dual retention mechanism can be stated by Eq. (9) (Farag et al., 2007; Han et al., 2017):

$$C_{r}=C_{\text{abs}}+C_{\text{ads}}=DC_{\text{aq}}+\frac{SK_{L}C_{\text{aq}}}{1+K_{L}C_{\text{aq}}}$$ (9)

where C_r_ and C_aq_ are the equilibrium concentrations of the tested EY on the solid sorbent and in aqueous solution, respectively. The parameters C_abs_ and C_ads_ are the equilibrium concentrations of the EY on the tested solid sorbents as an absorbed and adsorbed species, respectively, and S and K_L_ are the saturation values for Langmuir adsorption.

Additionally, surface complex formation of silanol and the electrostatic interaction of the Sol gel and the dye anion may also participate in the efficient performance of dye retention. The Sol-gel coating also has a positive impact on dye retention via H-bonding between the silanol group of the Sol gel and the dye anion, in addition to the electrostatic π-π interactions between the dye and the silicon/zirconium oxide (Si-O-Zr) and siloxane (Si-O-Si) groups of the Sol gel and PUFs (Olu-owolabi et al., 2014). Thus, the Sol–gel coating technology enhanced the porous structure of the PUFs, resulting in an improvement of the extraction efficiency of the dye. The increased dye retention at low pH (<3) suggested that the initial hydrophilic species located at the PUF surface tended to transform into hydrophobic species via polycondensation of residual hydroxyl Si-OH and Zr-OH into hydrophobic Si-O-Si and Si-O-Zr groups of the Sol gel.

### 3.7 Analytical applications

Validation of the proposed methodology was performed using recovery tests with known concentrations of the EY dye spiked into environmental water samples (500.0 mL) and adjusted to the optimized pH for dye sorption. The results of EY dye uptake from aqueous media at the optimized analytical parameters are summarized in Table 1. An acceptable retention of the EY dye was achieved. The retained EY dye species in the Sol-gel/PUFs were then quantitatively ( 92.0± 0.2–99.31 ± 0.1) recovered with NaOH, affirming the effectiveness of the analytical utility of the Sol gel sorbent for dye removal from water samples. The obtained *Student t* test values (2.3–2.6) at 95% confidence were found to be less than the theoretical *Student t* test values (2.78, *n* = 5), and the average recovery of the spiked samples ranged from 92.0± 0.2–99.31 ± 0.1, with an RSD less than ±5.0%. These data added further validation to the accuracy and precision of the developed Sol gel/PUFs extractor. The obtained data for the Sol-gel/PUFs showed that it can be used in a packed mini column for complete extraction and recovery of EY dye from environmental water samples using the batch mode of separation.

**TABLE 1:** Analytical results for the extraction and recovery of Eosin Y dye spiked in tap water samples using the proposed Sol-gel/PUFs batch mode.

Sample	Eosin Y dye added, μg/mL	Eosin Y dye found, μg/mL	Recovery, %
Tap water	20	19.86 ± 0.01	99.31 ± 0.1
	10	9.36 ± 0.01	93.65 ± 0.2
	5	4.604± 0.01	102.28± 0.01

### 3.8 Reusability of the established solid platform

Under optimal conditions, the reusability of the established solid extractor for EY sorption from aqueous solutions was tested as follows: after each batch experiment, the SPE was collected, washed several times with deionized water and acetone, dried at room temperature between filter paper, and re-packed into the mini column for the extraction process. After three sorption-desorption experiments, the adsorption capacity of the EY dye still retains *>*92 ± 0.2%, showing its good reusability, without a significant decrease in analyte uptake or stripping by the established solid phase micro-extractor. The reusability of the established extractor makes it a competent sorbent for dye uptake from aqueous media, demonstrating its clear, relevant, and practical implications.

## 4 Conclusion, advantages, and future challenges

### 4.1 Conclusion, advantages, and limitations

In summary, the current study provides simple and potentially low-cost Sol-gel impregnated polyurethane foams as d-µ SPME, for the first time, for the complete removal and subsequent trace spectrophotometric determination of EY dye in water. Based on the synergistic interplay between the Sol gel and the porous PUF membrane-like structures, the advanced functional materials developed display good performance toward the detection and removal of textile dyes compared to conventional materials. The unique structures of the PUFs and Sol gel materials complement the improved stability and environmental sustainability of the sorbent. Through an in-depth study on dye retention by the developed extractor, it is evident that a wide range of sophisticated platforms have been developed to meet the increasing demand for efficient separation and detection of the dye. The Sol-gel/PUFs solid platform combines the advantages of flexibility, good hydrodynamic and aerodynamic properties, high surface area, surface functionality, and selectivity. A proposed mechanism of EY dye sorption involving both “surface adsorption” and an added component of “ion exchanger and/or solvent extraction,” in addition to H-bonding between the silanol groups of the Sol-gel and the target dye is demonstrated. Other approaches, such as a microbial approach to dye-containing wastewater remediation have high operating costs, are less effective, and sometimes produce undesirable by-products when compared to physical and chemical methods, and should be administered over a long period of time. The innovative and cutting-edge tools reported in the current study have managed to showcase an extraordinary growth in the design of functional materials that are not only suitable, stable, and rapid, but also sensitive, eco-friendly, and recyclable.

### 4.2 Future perspectives

The combination of Sol gel-based porous substrates, such as polymers and porous materials, shows significant potential, and while remarkable progress has been made for the treatment of dye contamination, extraction systems are still at an early stage and critical trials remain to be performed, which limits their further utility in real-world applications. Dye detection and removal systems based on functional materials still need to be improved. A “future perspective” and potential recommendations for work includes: i) enhancing the sensing and extraction ability and facilitating detection in drinking water; ii) further developments and innovations in the Sol-gel microextraction phases are expected in the near future, involving new substrates, e.g., bio and magnetic materials; iii) synthesis and characterization of innovative and highly porous functional hybrid Sol-gel sorbent solid phase extractors; and iv) the use of experimental design is highly recommended in future work, since, in the proposed approach, the number of parameters is large and a step-by-step approach does not provide the interactions among the trial parameters. The potential utility of Sol-gel/PUFs for water treatment and the significant expansion of Sol-gel/PUFs applications for the development of innovative spectrochemical sensing strategies for dye determination are also highlighted. The specific modification strategy described here might necessitate further modification for large-scale production of the demonstrated analyte sorption.

## Data Availability

The original contributions presented in the study are included in the article/Supplementary material, further inquiries can be directed to the corresponding author.
